# Associations Between Coffee Consumption and the Prevalence of Metabolic Syndrome: A Nationwide Cross-Sectional Survey of Taiwanese Adults

**DOI:** 10.3390/nu18030463

**Published:** 2026-01-30

**Authors:** Ping-Yi Kuo, Jiun-Hung Geng, Pei-Yu Wu, Jiun-Chi Huang, Szu-Chia Chen

**Affiliations:** 1 Department of Medicine, Kaohsiung Medical University, Kaohsiung 807, Taiwan; kuopin0404@gmail.com; 2 Department of Urology, Kaohsiung Medical University Hospital, Kaohsiung Medical University, Kaohsiung 807, Taiwan; u9001090@hotmail.com; 3 Department of Urology, Kaohsiung Municipal Siaogang Hospital, Kaohsiung Medical University Hospital, Kaohsiung Medical University, Kaohsiung 812, Taiwan; 4 School of Medicine, College of Medicine, Kaohsiung Medical University, Kaohsiung 807, Taiwan; wpuw17@gmail.com (P.-Y.W.); karajan77@gmail.com (J.-C.H.); 5 Department of Internal Medicine, Kaohsiung Municipal Siaogang Hospital, Kaohsiung Medical University Hospital, Kaohsiung Medical University, Kaohsiung 812, Taiwan; 6 Division of Nephrology, Department of Internal Medicine, Kaohsiung Medical University Hospital, Kaohsiung Medical University, Kaohsiung 807, Taiwan

**Keywords:** coffee consumption, metabolic syndrome, metabolic syndrome components, Taiwan biobank

## Abstract

**Background/Objectives:** Findings on the association between metabolic syndrome (MetS) and coffee consumption are conflicting. **Methods:** This cross-sectional study included a large Taiwanese cohort and aimed to investigate associations between coffee consumption and the risk of MetS and individual components of MetS. Data of 27,119 participants (17,530 females and 9589 males; mean age 55.0 ± 10.3 years) were obtained from the Taiwan Biobank from July 2011 to November 2019. Associations among coffee consumption (type, intake and frequency) with MetS and its components were examined with multivariable logistic regression analysis, which included the significant variables in univariable analysis. Coffee consumption was assessed according to frequency, type and intake. **Results:** The results showed an association between coffee consumption and a lower risk of MetS (odds ratio [OR], 0.875; *p* < 0.001). Significant associations were found between the consumption of black coffee (OR, 0.848; *p* < 0.001) and coffee with milk (OR, 0.848; *p* = 0.001) with a low risk of MetS, while coffee with creamer was not. Daily consumption of one or two cups (237–474 mL) (OR, 0.805; *p* < 0.001 and 0.887; *p* = 0.001, respectively) was significantly associated with a low prevalence of MetS, whereas daily consumption of three or more cups was not. In addition, the participants who drank coffee every day (OR, 0.811; *p* < 0.001) were significantly associated with a low prevalence of MetS, whereas those who only drank coffee weekly or monthly were not. Further, significant associations were found between coffee consumption with lower risks of hypertriglyceridemia (OR, 0.844; *p* < 0.001) and low high-density lipoprotein cholesterolemia (OR, 0.836; *p* < 0.001) but not with abdominal obesity, hyperglycemia or high blood pressure. **Conclusions:** The regular consumption of black coffee or coffee with milk was linked to a low prevalence of MetS and certain components. Longitudinal studies are warranted to confirm these findings and elucidate the underlying mechanisms.

## 1. Introduction

Metabolic syndrome (MetS) is defined as the co-occurrence of metabolic risk factors including a high level of triglycerides, low high-density lipoprotein (HDL) cholesterol, hyperglycemia, abdominal obesity, and hypertension, and it is associated with increased risks of cardiovascular disease, type 2 diabetes, and mortality [1]. The global prevalence of MetS in adults is estimated to range from 20 to 25% [2]. The pathophysiology of MetS is complex and involves interactions between genetic, environmental, and lifestyle factors [3]. Insulin resistance is considered a central mechanism [4], leading to impaired glucose uptake, compensatory hyperinsulinemia, and altered hepatic lipid handling. Visceral adiposity is now recognized as an active endocrine organ that secretes pro-inflammatory adipokines such as tumor necrosis factor (TNF)-α, interleukin (IL)-6, and resistin while downregulating protective adipokines like adiponectin [5]. This imbalance promotes chronic low-grade inflammation, endothelial dysfunction and further insulin resistance [6]. Excess free fatty acids released from visceral fat contribute to hepatic steatosis, increased triglyceride-rich lipoprotein synthesis, and reduced HDL levels, thereby fostering atherogenic dyslipidemia [7]. In addition, activation of the renin-angiotensin–aldosterone system, sympathetic overactivity, oxidative stress, and mitochondrial dysfunction contribute to hypertension and metabolic impairment [8]. Physical inactivity, unhealthy dietary patterns, excessive alcohol intake, smoking, and genetic predisposition further exacerbate metabolic dysregulation [9,10]. Given the significant rise in the global prevalence of MetS, effective prevention and management strategies have become increasingly important [11].

Coffee is a very popular beverage and consumed worldwide, and its health effects have been widely studied. Regarding the negative effects of coffee consumption, some evidence has shown an association with insomnia [4], while evidence of its relationship with gastroesophageal reflux disease [12] and elevated blood pressure [13] remains inconclusive. Moreover, substantial evidence suggests that pregnant and postmenopausal women should avoid excessive coffee consumption, as caffeine may interfere with oral contraceptives or postmenopausal hormone therapy [4]. However, several review articles have indicated that coffee may have health benefits, such as enhancing metabolism [14], reducing glucose absorption [15], and reducing the risk of Alzheimer’s disease [4]. The literature examining the association between coffee consumption and MetS has yielded inconsistent findings across longitudinal, cross-sectional, and meta-analytic studies [16,17,18,19]. These discrepancies may reflect heterogeneity in coffee preparation (e.g., black, milk-based, creamer-based), consumption patterns across cultures, and population-specific metabolic profiles. Moreover, Asian populations exhibit dietary and metabolic characteristics distinct from Western cohorts, yet few studies have simultaneously evaluated coffee type, intake, and frequency in this region. Therefore, we aimed to clarify these relationships using a large Taiwanese cohort.

The potential mechanisms underlying the association between MetS and coffee consumption may involve bioactive compounds found in coffee such as chlorogenic acid, caffeine, and polyphenols [20], as these compounds are known to influence lipid metabolism [21] and inflammatory responses [22]. Specifically, chlorogenic acids can inhibit hepatic glucose-6-phosphatase and delay intestinal glucose absorption, thereby improving insulin sensitivity [23]. Polyphenols enhance fatty acid oxidation and suppress hepatic de novo lipogenesis, leading to reduced triglyceride synthesis and improved HDL concentrations [24]. Coffee bioactives can also modulate adipokine secretion, increasing adiponectin and reducing pro-inflammatory cytokines such as TNF-α and IL-6, which attenuates chronic low-grade inflammation and endothelial dysfunction, which are two central features of MetS [25]. However, findings from previous studies on this topic remain inconsistent. While a longitudinal study reported a protective effect of coffee against MetS [26], another cross-sectional study suggested an increased risk [16], and several meta-analyses reported no significant associations [17,18]. Although coffee consumption has been widely examined in relation to metabolic health, most existing studies have been conducted in Western populations and have not simultaneously evaluated different aspects of consumption, such as coffee type, intake, and frequency, in relation to MetS and its individual components. Moreover, Asian populations exhibit distinct metabolic profiles, dietary behaviors, and coffee consumption patterns, including the common use of milk- or creamer-based beverages, which may lead to metabolic effects that differ from those observed in Western cohorts. Given the rising prevalence of MetS in East Asian countries, population-specific evidence on modifiable dietary behaviors is increasingly important for prevention and public health planning. Therefore, we conducted this study using a large cohort from the Taiwan Biobank to clarify the associations between coffee consumption (including type, intake, and frequency) and MetS and its components, and to address an important gap in the current literature.

## 2. Materials and Methods

### 2.1. Study Participants

To enhance biomedical and epidemiological research in Taiwan, the TWB was launched by the government in 2012 as an ongoing prospective study of males and females aged 30–70 years recruited from approximately 30 centers around the country. The TWB collects health-related data on healthy volunteers around Taiwan. None of the enrollees in the TWB have a history of cancer. Comprehensive genomic and phenotypic data are collected and recorded for each participant at enrollment and during follow-up visits through structured questionnaires, physical examinations, and urine and blood tests [19,27]. The TWB was established by the Ministry of Health and Welfare to improve healthcare for the residents of Taiwan, and it was approved by the Ethics and Governance Council of the TWB and Institutional Review Board (IRB) on Biomedical Science Research at Academia Sinica. All participants in the TWB provided written informed consent before enrollment.

**Figure 1:**
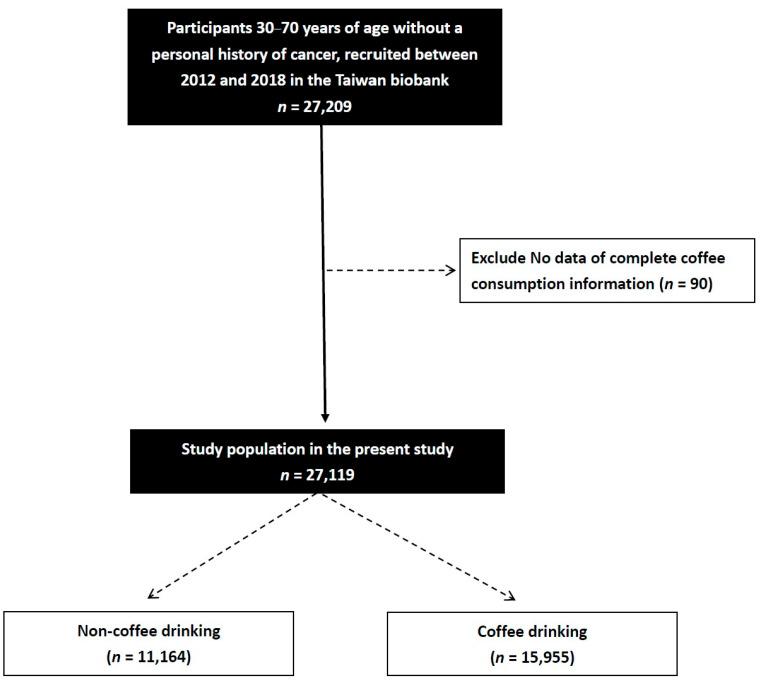
Flowchart of study population.

We obtained data on 27,209 participants (17,530 females and 9589 males; mean age 55.0 ± 10.3 years) from the Taiwan Biobank (TWB). We excluded 90 patients who did not have information on coffee consumption, and the remaining 27,119 were enrolled in this cross-sectional study (Figure 1).

After participants signed informed consent forms, they were interviewed and underwent physical and blood tests. During these examinations, data on tobacco and alcohol use, presence of hypertension and diabetes mellitus [DM], sex, age, height, weight, hip and waist circumference (WC) are obtained. In addition, data on HDL/LDL and total cholesterol, triglycerides, fasting glucose, uric acid and hemoglobin are also recorded, along with estimated glomerular filtration rate (eGFR) calculated with the creatinine equation of the Chronic Kidney Disease Epidemiology Collaboration 2021 [28]. Fasting blood samples were obtained from all of the patients, and laboratory tests were conducted using an autoanalyzer (Roche Diagnostics GmbH, D-68298, COBAS Integra 400, located in Mannheim, Germany). Regular exercise was defined as performing any kind of physical activity for ≥30 min ≥3 times a week [29].

Blood pressure (systolic/diastolic [SBP/DBP]) was recorded by personnel of the TWB following standard procedures, including a 1 to 2 min gap between readings and the avoidance of smoking, caffeine, and physical activity for a minimum of 30 min. The averages of three readings were used in the analysis. The level of physical activity was also recorded, with regular activity defined as at least three 30 min sessions a week [29]. This study complied with the principles established in the Declaration of Helsinki, and the IRB of Kaohsiung Medical University Hospital granted ethical approval (KMUHIRB-E(I)-20210058, 23 March 2023).

### 2.2. Definition of DM and Hypertension

Participants with past history of DM (self-reported), with a fasting glucose level ≥ 126 mg/dL and HbA1c ≥ 6.5% were defined as having DM.

Hypertension was defined as participants who were already using antihypertensive medications or had a SBP ≥ 140 mmHg or DBP ≥ 90 mmHg.

### 2.3. Assessment of Alcohol Drinking and Cigarette Smoking History

The definitions of habits for alcohol drinking and cigarette smoking history were as follows. Those who had smoked 1 cigarette per day for at least 1 year were defined as ever smokers. For alcohol drinking, those who had consumed any alcoholic beverage > 4 times a week for at least 1 year were defined as ever drinkers.

### 2.4. Assessment of Coffee Consumption

Coffee consumption was assessed by asking the participants if they regularly drank coffee. The participants were then classified into coffee drinker and non-coffee drinker groups accordingly. The coffee drinkers were then asked the following questions:

1. “What type of coffee do you usually drink?” The participants were then classified into the following groups accordingly: “black coffee”, “coffee with creamer” or “coffee with milk”.
2. “How many cups of coffee (one cup = 237 mL) do you typically consume daily?” The participants were then classified into “none”, “one cup per day”, “two cups per day” or “three cups or more per day” groups.
3. “How often do you drink coffee?” The participants were then classified into “never”, “every day”, “weekly” (frequency less than every day), and “monthly” (frequency less than weekly) groups.

### 2.5. Definition of MetS

A diagnosis of MetS was ascertained following the NCEP-ATP III guidelines modified for an Asian population [30] as the presence of ≥3 MetS components, defined as: (1) fasting glucose level ≥ 110 mg/dL or a diagnosis of DM; (2) triglyceride level ≥ 150 mg/dL; (3) HDL cholesterol < 50/40 mg/dL for women/men; (4) SBP/DBP ≥ 130/85 mmHg, a diagnosis of hypertension, or prescription for anti-hypertension medications; (5) abdominal obesity (WC > 80/90 cm for women/men).

### 2.6. Statistical Analysis

Data were given in percentage or mean ± SD. Continuous variables were compared using the independent *t* test, and categorical variables were compared using the chi-square test. Associations among coffee consumption (type, intake and frequency) with MetS and its components were examined using multivariable logistic regression analysis, which included the significant variables in univariable analysis. *p* values < 0.05 were considered statistically significant. The statistical analysis was performed with SPSS v25 for Windows^®^ (IBM Inc., Armonk, NY, USA).

## 3. Results

### 3.1. Clinical Characteristics of the Participants with and Without MetS

Of the 27,119 enrolled participants, 7171 (26.4%) had MetS and 19,948 (73.6%) did not; their clinical characteristics are presented in Table 1. Compared to the non-MetS group, more of the MetS group were male and coffee drinkers. In addition, they were older, had higher rates of DM, hypertension, tobacco and alcohol use, and higher SBP/DBP, weight, WC, hip circumference, fasting glucose, hemoglobin, triglycerides and uric acid compared to those without MetS, along with lower total cholesterol, HDL cholesterol, LDL cholesterol, and eGFR. Regarding the components of MetS, the MetS group had higher levels of abdominal obesity, hypertriglyceridemia, low HDL cholesterolemia, hyperglycemia and hypertension.

### 3.2. Associations Between Coffee Consumption and MetS

Multivariable logistic regression analysis was performed adjusting for the significant variables shown in Table 1 (age, sex, total and LDL cholesterol, tobacco and alcohol use, coffee drinkers, uric acid, hemoglobin and eGFR). The analysis revealed that female sex, older age, smoking history, alcohol history, non-coffee drinkers (odds ratio [OR], 0.875; 95% confidence interval [CI], 0.825–0.928; *p* < 0.001), high hemoglobin, low total cholesterol, and high uric acid were significantly associated with a high prevalence of MetS (all *p* < 0.001) (Table 2).

### 3.3. Associations Among the Type, Intake and Frequency of Coffee Consumption with MetS

Multivariable logistic regression analysis after adjusting for the significant variables in Table 1 showed that those who drank black coffee (vs. non-coffee drinkers; OR, 0.848; 95% CI, 0.791–0.910; *p* < 0.001), and coffee with milk (vs. non-coffee drinkers; OR, 0.848; 95% CI, 0.766–0.938; *p* = 0.001) were significantly associated with a low prevalence of MetS, while those who drank coffee with creamer (*p* = 0.567) were not (Table 3).

The ORs for MetS according to coffee consumption frequency are also shown in Table 3. Compared to those who did not regularly consume coffee (no cups per day), those who drank one cup per day (OR, 0.805; 95% CI, 0.734–0.883; *p* < 0.001), and two cups per day (OR, 0.887; 95% CI, 0.827–0.951; *p* = 0.001) were significantly associated with a low prevalence of MetS, while those who drank three cups or more per day (*p* = 0.520) were not.

Regarding the frequency of drinking coffee, the participants who drank coffee every day (OR, 0.811; 95% CI, 0.756–0.870; *p* < 0.001) were associated with a significantly lower prevalence of MetS compared to those who did not regularly drink coffee. However, those who drank coffee weekly (*p* = 0.532) or monthly (*p* = 0.511) were not significantly associated with MetS.

### 3.4. Associations Between Coffee Consumption and Individual Components of MetS

Multivariable logistic regression analysis after adjusting for the significant variables in Table 1 showed significant associations between coffee consumption with hypertriglyceridemia (OR, 0.844; 95% CI, 0.792–0.899; *p* < 0.001) and low-HDL cholesterolemia (OR, 0.836; 95% CI, 0.788–0.887; *p* < 0.001) but not with abdominal obesity (*p* = 0.647), hyperglycemia (*p* = 0.337), or hypertension (*p* = 0.464) (Table 4).

## 4. Discussion

The results of this study showed an association between drinking black coffee and coffee with milk with a low prevalence of MetS, whereas drinking coffee with creamer showed no significant benefit. In addition, the daily consumption of 237 mL (one cup) or 474 mL (two cups) of coffee was also associated with a low prevalence of MetS, while drinking more than three cups a day showed no significant association. Furthermore, the protective effect was observed only among individuals who consumed coffee daily and not in those who drank coffee weekly or monthly. Further analysis revealed an association between coffee consumption and lower risks of low HDL cholesterol and hypertriglyceridemia; however, this association was not found for abdominal obesity, hyperglycemia, or hypertension.

The first important finding is that drinking black coffee or coffee with milk was associated with a low prevalence of MetS. This finding is consistent with the study by Choi and Je [31] who investigated the association between coffee consumption including the type of coffee and MetS using data from the Korea National Health and Nutrition Examination Survey conducted from 2016 to 2021. The study included 14,631 adults aged 19–64 years, and the results showed that women who drank two to three cups of black coffee a day were inversely associated with MetS (OR = 0.66; 95% CI = 0.46–0.96), whereas the participants who drank coffee with sugar or cream or more than three cups per day were not significantly associated with MetS [31]. Another study in Taiwan found that only men with a daily coffee consumption of more than one cup or individuals who drank black coffee were associated with a lower prevalence of MetS. Moreover, women who drank any amount of coffee and any type of coffee were associated with a significantly lower prevalence of MetS than those who did not drink coffee [32]. Our results are broadly consistent with prospective cohort studies and meta-analyses reporting inverse associations between moderate coffee consumption and MetS incidence [16,17,18,19]. However, previous pooled analyses rarely stratified by coffee type, intake, or frequency, and often relied on Western dietary cohorts. By demonstrating that black coffee and milk-based coffee, but not creamer-based coffee, were inversely associated with MetS in a large Asian cohort, our findings refine existing evidence and suggest that preparation methods and cultural consumption patterns may partly explain heterogeneity across studies. Previous studies have suggested that chlorogenic acid and polyphenols in coffee may influence MetS risk by regulating lipid metabolism [21,33], exerting an anti-inflammatory effect [22,33,34], and improving insulin sensitivity [35,36]. In addition, other studies have suggested that coffee may reduce MetS risk by inhibiting intestinal glucose absorption [37,38] and exerting antioxidant effects [39,40]. Notably, black coffee and unsweetened milk-based coffee appeared to have a stronger protective effect, whereas coffee with creamer or added sugar did not show significant benefits [31]. This may be because sugar and creamer interfere with the bioavailability of chlorogenic acid in the human body, whereas milk does not [41]. The potential mechanisms by which black coffee or coffee with milk may confer metabolic benefits likely involve the bioactive compounds of coffee and the compositional differences among beverage types. Black coffee provides chlorogenic acids and polyphenols without added sugars or saturated fats, which may enhance lipid metabolism, improve insulin sensitivity, and reduce low-grade inflammation through antioxidant and anti-inflammatory pathways [23,24]. Milk-based coffee without added sugar may retain these benefits while providing dairy proteins and calcium, which have been reported to exert favorable effects on weight regulation, lipid metabolism, and glucose homeostasis in some studies. In contrast, the addition of creamer or sugar may attenuate these mechanisms by increasing caloric load, promoting insulin resistance, and diminishing the bioavailability of chlorogenic acids [42], thereby offering a plausible explanation for the differential metabolic effects observed across coffee types.

The second important finding is that daily regular consumption of one to two cups (237–474 mL) of coffee was associated with a lower risk of MetS, while consuming more than three cups per day did not show a significant protective effect. This may suggest that the quantity of coffee consumption also plays a crucial role in reducing the risk of MetS. Similarly, the results of a previous study examining the association between coffee consumption and MetS showed that, in women, consuming two to three cups a day of black coffee was inversely associated with MetS but that consuming coffee with sugar or cream or daily consumption of more than three cups was not significantly associated with MetS in the whole cohort [31]. In a Mediterranean cohort of 10,253 individuals who were initially free of MetS and then evaluated after 6 years of follow-up, 398 developed MetS. Further analysis showed that daily consumption of one to three cups of coffee was associated with a significantly lower risk of developing MetS compared to consuming less than one cup a month [26]. However, long-term high coffee consumption has been suggested to increase the risk of cardiovascular disease, possibly because such consumption may lead to hyperlipidemia, a well-established risk factor for cardiovascular disease [43]. In addition, high caffeine intake may cause anxiety, insomnia, tremulousness, and palpitations, as well as bone loss and possibly increase the risk of fractures [44]. One plausible explanation is that high-volume coffee consumption may more frequently involve beverages containing added sugar or creamer, which could attenuate the metabolic benefits of coffee. In addition, excessive caffeine intake may negatively affect sleep quality, blood pressure regulation, and stress responses in susceptible individuals, thereby counteracting the beneficial effects observed at moderate intake levels. Further research is needed to clarify the threshold between adequate and excessive coffee consumption and its effects on physical health.

We also found that only daily coffee consumption was beneficial in reducing MetS risk, whereas weekly or monthly consumption did not confer significant benefits. This may be related to the half-life of caffeine, as its physiological effects may become negligible without continuous intake [45]. Coffee is thought to influence MetS primarily through the regulation of lipid metabolism [21,33], anti-inflammatory effects [22,33,34], and improvements in insulin sensitivity [35,36], all of which require long-term consumption to be effective. However, these associations may also reflect alternative explanations. Consumption frequency may be a proxy for broader lifestyle or dietary co-patterns, whereby daily coffee drinkers engage in healthier behaviors. Residual confounding due to unmeasured diet quality, socioeconomic status, or physical activity cannot be excluded. Reverse causation is also possible if individuals with metabolic abnormalities reduce their coffee intake. Given the cross-sectional design, causal inference cannot be established. This may explain why only daily consumption has been shown to have a significant impact.

Another key finding is the association between coffee consumption and lower risks of low HDL cholesterol and hypertriglyceridemia. A previous Korean study assessed the association between coffee consumption and MetS, and found that, compared to non-coffee drinkers, males with moderate consumption (≤3 cups/day) had lower risks of low HDL cholesterol and high fasting blood glucose [46]. In addition, the male participants who drank 3-in-1 coffee (which included creamer and sugar) also had lower risks of low HDL cholesterol and high fasting blood glucose. In a systematic review and meta-analysis conducted in Korea, analysis of pooled effect sizes from at least four individual study populations showed a significant association between coffee consumption and high triglycerides (OR, 0.84; 95% CI, 0.78–0.90) [47]. Choi and Je also found that, of the individual components of MetS, coffee consumption had a more pronounced effect on low HDL cholesterol and hypertriglyceridemia [31], possibly due to the regulatory effects of the bioactive compounds of coffee on lipid metabolism. However, from a review article and a meta-analysis, coffee consumption did not have a significant effect on abdominal obesity, hyperglycemia, and hypertension [48,49], suggesting that the influence of coffee on MetS may be selective and not uniformly affect all MetS components. These bioactive compounds may selectively influence lipid metabolism pathways more strongly than other MetS components. Chlorogenic acids and polyphenols have been shown to enhance fatty acid oxidation, downregulate hepatic lipogenesis, and inhibit very-low-density lipoprotein secretion, thereby lowering circulating triglycerides while improving HDL concentrations [23,24]. In contrast, their effects on blood pressure, fasting glucose, and abdominal obesity appear to be more modest or indirect, which may help explain why coffee consumption showed more pronounced associations with HDL and triglycerides in our analysis.

The inclusion of a large community-based sample of healthy individuals and controlling for confounders are important strengths of this study. However, there are also several limitations. First, the temporal association between coffee consumption and MetS could not be evaluated due to the cross-sectional design. Longitudinal investigations are required to validate our results. Second, the TWB database does not contain information on prescriptions for drugs that could influence hypertension, fasting glucose, lipid profiles, and obesity. Consequently, this may have affected our results regarding the association between coffee consumption and MetS. Third, we lack the information on the dietary patterns of participants, as diet can significantly influence both coffee consumption habits and metabolic syndrome risk. Fourth, information on coffee consumption relied on self-reported questionnaires, and recall bias is possible. Fifth, we did not assess the maximum amount of coffee intake that could effectively lower the risk of MetS, because some people may experience side effects related to the bioactive compounds. Finally, more female participants are enrolled in the TWB than men, possibly because women are generally more willing to volunteer for research studies, and thus our findings may not be generalizable to the general population.

## 5. Conclusions

In conclusion, this study demonstrated that coffee consumption was associated with a lower prevalence of MetS, particularly when consuming black coffee or coffee with milk. In addition, we found that drinking one to three cups of coffee per day was beneficial in lowering the risk of MetS but that this effect was only observed in those who drank coffee daily. Moreover, coffee intake was associated with a low prevalence of low HDL cholesterol and hypertriglyceridemia. This cross-sectional study suggests that drinking 1–3 cups of black coffee or coffee with milk daily might lower the risk of MetS by improving HDL cholesterol and triglycerides. Further longitudinal studies are needed to confirm these findings and elucidate the underlying mechanisms. More precise analysis is needed, including usual dietary habits, the precise type and amount of coffee, so that we can better explain the cause-and-effect relationship.

## Figures and Tables

**Table 1:** Comparisons of the clinical characteristics between the participants with and without MetS.

Characteristics	MetS (−) (*n* = 19,948)	MetS (+) (*n* = 7171)	*p*
Age (year)	54.0 ± 10.4	57.8 ± 9.6	<0.001
Male sex (%)	33.8	39.6	<0.001
DM (%)	4.0	18.1	<0.001
Hypertension (%)	10.7	37.9	<0.001
Smoking history (%)	23.9	30.2	<0.001
Alcohol history (%)	9.6	13.5	<0.001
Coffee consumption (%)	42.1	28.4	<0.001
Regular exercise habits (%)	48.5	47.7	0.278
Systolic BP (mmHg)	120.8 ± 18.3	136.0 ± 18.2	<0.001
Diastolic BP (mmHg)	72.2 ± 10.8	79.8 ± 11.2	<0.001
Body height (cm)	160.7 ± 8.0	160.9 ± 8.5	0.143
Body weight (kg)	60.6 ± 10.7	70.1 ± 12.7	<0.001
Waist circumference (cm)	81.3 ± 8.8	91.4 ± 8.9	<0.001
Hip circumference (cm)	94.4 ± 6.3	99.1 ± 7.4	<0.001
Laboratory parameters			
Fasting glucose (mg/dL)	92.9 ± 14.4	109.8 ± 31.6	<0.001
Hemoglobin (g/dL)	13.6 ± 1.5	14.1 ± 1.5	<0.001
Triglyceride (mg/dL)	94.7 ± 51.5	190.8 ± 146.2	<0.001
Total cholesterol (mg/dL)	196.5 ± 35.2	195.3 ± 38.9	0.023
HDL-C (mg/dL)	58.1 ± 12.9	44.6 ± 9.6	<0.001
LDL-C (mg/dL)	120.4 ± 31.0	119.4 ± 34.1	<0.001
eGFR (mL/min/1.73 m^2^)	99.5 ± 14.2	93.9 ± 16.7	0.030
Uric acid (mg/dL)	5.2 ± 1.3	6.0 ± 1.4	<0.001
MetS component			
Abdominal obesity (%)	36.9	87.3	<0.001
Hypertriglyceridemia (%)	8.5	61.9	<0.001
Low HDL cholesterol (%)	12.4	65.3	<0.001
Hyperglycemia (%)	12.2	58.1	<0.001
High BP (%)	31.6	79.5	<0.001

Abbreviations. BP, blood pressure; DM, diabetes mellitus; eGFR, estimated glomerular filtration rate; HDL-C, high-density lipoprotein cholesterol; LDL-C, low-density lipoprotein cholesterol; MetS, metabolic syndrome.

**Table 2:** Association between coffee consumption and MetS in multivariable regression analysis.

Variables	Multivariable (MetS)	
	OR (95% CI)	*p*
Age (per 1 year)	1.037 (1.033–1.040)	<0.001
Male sex (vs. female)	0.370 (0.337–0.406)	<0.001
Smoking history	1.286 (1.186–1.395)	<0.001
Alcohol history	1.118 (1.015–1.232)	0.024
Coffee consumption	0.875 (0.825–0.928)	<0.001
Laboratory parameters		
Hemoglobin (per 1 g/dL)	1.278 (1.246–1.312)	<0.001
Total cholesterol (per 1 mg/dL)	0.997 (0.996–0.999)	0.002
LDL-C (per 1 mg/dL)	0.998 (0.997–1.000)	0.124
eGFR (per 1 mL/min/1.73 m^2^)	1.001 (0.998–1.004)	0.484
Uric acid (per 1 mg/dL)	1.589 (1.549–1.630)	<0.001

For the abbreviations see Table 1.

**Table 3:** Associations among the content, daily cups and frequency of coffee consumption with MetS in multivariable regression analysis.

Coffee Consumption	Multivariable (MetS)	
	OR (95% CI)	*p*
Coffee content		
Non-coffee	Reference	
Black coffee	0.848 (0.791–0.910)	<0.001
Coffee with creamer	1.035 (0.919–1.166)	0.567
Coffee with milk	0.848 (0.766–0.938)	0.001
Daily cups		
None	Reference	
1 cup ∗ per day	0.805 (0.734–0.883)	<0.001
2 cups ∗ per day	0.887 (0.827–0.951)	0.001
≥3 cups ∗ per day	1.049 (0.907–1.214)	0.520
Frequency		
None	Reference	
Per day	0.811 (0.756–0.870)	<0.001
Per week	0.975 (0.900–1.056)	0.532
Per month	1.459 (0.473–4.498)	0.511

For the abbreviations see Table 1. Adjusted for age, sex, tobacco and alcohol history, hemoglobin, total cholesterol, LDL cholesterol, eGFR and uric acid. ∗ One cup = 0.237 L.

**Table 4:** Associations between coffee consumption and individual components of MetS in multivariable logistic regression analysis.

Variables	Abdominal Obesity		Hypertriglyceridemia		Low HDL Cholesterol		Hyperglycemia		High BP	
	OR (95% CI)	*p*	OR (95% CI)	*p*	OR (95% CI)	*p*	OR (95% CI)	*p*	OR (95% CI)	*p*
Coffee consumption	1.012 (0.961–1.066)	0.647	0.844 (0.792–0.899)	<0.001	0.836 (0.788–0.887)	<0.001	1.030 (0.970–1.093)	0.337	0.980 (0.928–1.034)	0.464

For the abbreviations see Table 1. Adjusted for age, sex, tobacco and alcohol history, hemoglobin, total cholesterol, LDL cholesterol, eGFR and uric acid.

## Data Availability

The data presented in this study are available on request from the corresponding author. The data are not publicly available due to restrictions placed on the data by the Personal Information Protection Act of Taiwan. Data may be available upon request to interested researchers.
